# A Novel Three-Point Localization Method for Bladder Volume Estimation

**DOI:** 10.3390/s24061932

**Published:** 2024-03-18

**Authors:** Junru Yuan, Mingke Shen, Tao Zhang, Jun Ou-Yang, Xiaofei Yang, Benpeng Zhu

**Affiliations:** School of Integrated Circuits, Huazhong University of Science and Technology, Wuhan 430074, China

**Keywords:** bladder volume estimation, triple-element ultrasound probe, three-point localization method

## Abstract

The measurement of bladder volume is crucial for the diagnosis and treatment of urinary system diseases. Ultrasound imaging, with its non-invasive, radiation-free, and repeatable scanning capabilities, has become the preferred method for measuring residual urine volume. Nevertheless, it still faces some challenges, including complex imaging methods leading to longer measurement times and lower spatial resolution. Here, we propose a novel three-point localization method that does not require ultrasound imaging to calculate bladder volume. A corresponding triple-element ultrasound probe has been designed based on this method, enabling the ultrasound probe to transmit and receive ultrasound waves in three directions. Furthermore, we utilize the Hilbert Transform algorithm to extract the envelope of the ultrasound signal to enhance the efficiency of bladder volume measurements. The experiment indicates that bladder volume estimation can be completed within 5 s, with a relative error rate of less than 15%. These results demonstrate that this novel three-point localization method offers an effective approach for bladder volume measurement in patients with urological conditions.

## 1. Introduction

Urinary tract diseases are common among the elderly, with the incidence significantly increasing with age [1,2]. In addition, in gynecological treatments, procedures like hysterectomy involve removing ligaments and connective tissues linking the bladder to the uterus, leading to common post-operative complications such as urinary retention and incontinence. Clinical manifestations of urinary retention involve an excessive amount of urine in the bladder that cannot be voided voluntarily, resulting in bladder overdistension and permanent damage to the detrusor muscle. The traditional clinical approach to address this issue involves using urinary catheterization, where a catheter is inserted into the urethra to drain urine from the bladder and, simultaneously, the residual urine volume in the bladder is measured. However, during this process, patients experience significant discomfort and an increased risk of urinary tract infections. Urinary hesitancy, urinary retention, and urinary incontinence are significant factors contributing to urinary tract diseases [3,4]. Hence, the early estimation of bladder volume is especially crucial, serving as an integral component in treating urinary system diseases and assessing treatment effectiveness.

Bladder volume estimation methods include the implantable sensor method [5,6,7,8,9,10,11,12,13,14,15], the bioimpedance tomography method [16,17,18], and magnetic resonance imaging for external imaging [19,20]. Various implantable bladder monitoring systems have been reported to estimate volume based on different physical properties, such as resistance [8], strain [9], pressure [10], capacitance [11], magnetism [12], and transmission into neural activity [13]. Some of these implanted systems have incorporated wireless telemetry technology, employing various power supply methods, including rechargeable batteries [14], radiofrequency waves [15], and acoustic wave radioelectric power transmission. These wireless implanted bladder monitoring systems are promising, but issues such as biofouling, drift, and reduced sensitivity have not yet been addressed. Furthermore, the invasive nature of implanted sensors underscores the critical importance of reliability, as recalibration and replacement post-implantation pose significant challenges. Leonhardt et al. [16] proposed using electrical impedance tomography to estimate bladder volume. The average conductivity index derived from electrical impedance tomography images quantifies bladder filling. Li et al. [17] found a strong linear correlation between the average conductivity index and bladder volume, indicating the ability of electrical impedance tomography to differentiate bladder urine volume. Although bioelectrical impedance techniques are simple and non-invasive, unreliable skin-electrode contact, various factors causing impedance fluctuations, and the need to use numerous electrodes for positioning pose challenges in electrode placement and may cause discomfort. Magnetic resonance imaging for bladder volume monitoring offers the advantages of non-invasiveness and high-resolution imaging. Heavily T_2_-weighted turbo spin-echo sequences describe the total amount of fluid in the volume within a few seconds [19]. Heverhagen et al. [20] utilized a 1.0-Tesla magnetic resonance scanner (Magnetom Expert, Siemens, Erlangen, Germany) to acquire a T_2_-weighted sequence with a 7-s acquisition time in 30 healthy volunteers. A histogram algorithm was used to evaluate pre- and post-voiding image fluid volumes, and the bladder volume was calculated from the difference between the pre- and post-voiding image fluid volumes. The measured voided volume was 400 ± 33 mL, while magnetic resonance hydrometry yielded 390 ± 31 mL, confirming the viability of magnetic resonance imaging for evaluating bladder volume. Moreover, Ma et al. [21] introduced a novel approach based on geodesic active contour algorithms to segment the inner and outer boundaries of the bladder wall, which helps to enhance the accuracy of bladder volume estimation. Indeed, despite the higher resolution of magnetic resonance imaging, its high cost and complexity render it less preferred in most cases. In addition, the magnetic resonance imaging method is not suitable for patients with internal metallic medical devices such as pacemakers and artificial joints [22].

Ultrasound imaging has gained increasing attention for bladder volume measurement due to its unique advantages, including non-invasiveness, repeatability, and suitability for large-scale applications [23,24,25,26,27]. McLean et al. [28] employed a 2.25 MHz focused transducer for two-dimensional ultrasound gray scale imaging to estimate bladder volume. The width was obtained by displaying the maximum transverse diameter through the transverse scan, while the height and depth were acquired through a midline longitudinal scan. Subsequently, these three dimensions were multiplied together to derive the bladder volume. While this method is straightforward and convenient, its accuracy is limited by the restricted field of view and resolution, providing only a rough estimation of the bladder volume. With the development of the 3D transducer, 3D ultrasonic volume measurement has attracted the interest of researchers. Baek et al. [29] utilized the 3D US device, Accuvix V20, and its 3D convex probe to obtain three-dimensional images. Subsequently, they employed the planimetry method using the continuous cross-sectional areas of these three-dimensional images to measure the volume of the ultrasound images. Their research demonstrates that 3D ultrasound volume measurements are more accurate compared to 2D ultrasound measurements [29]. However, these 3D ultrasound probes and related equipment are bulky, expensive, and require extended testing periods, which restricts their widespread adoption in clinical and practical applications. Currently, some wearable miniaturized ultrasonic bladder volume measurement devices employ distinct data processing methods that do not imply the provision of images as output. Instead, they rely on calculating pulse echo attenuation and time of flight. This shift allows for a significant reduction in size and computational time. Niu et al. [30] designed and implemented an ultrasound bladder volume measurement system based on ultrasound echo measurement technology. This system utilizes a novel bladder volume algorithm, estimating bladder volume solely from ultrasound echoes obtained by one phased-array ultrasonic transducer. Measurements conducted in vitro on phantoms using this system demonstrated its accuracy for a bladder volume greater than 100 mL. This method utilizes an ultrasound array transducer to enhance computational accuracy, but it faces challenges such as complex circuitry and high power consumption since each transducer element requires its own transmitting and receiving circuits. Given this, we have investigated a novel non-imaging method, namely the “three-point localization” method, to address the complexities and inaccuracies associated with existing bladder volume calculation methods. This method models the bladder as a sphere, utilizing echo signals to obtain distances from the ultrasound transducer to the bladder in three directions and constructing a triangular prism [31,32]. The approach is relatively straightforward, employing a Field–Programmable Gate Array (FPGA) development platform for bladder volume measurement, thereby significantly reducing the size of the instrument and cutting costs. This method exhibits extensive potential applications in the field of wearable devices.

Here, we introduced a novel bladder volume method known as the three-point localization method. Furthermore, the three-element ultrasound probe was designed based on our innovative bladder volume estimation method. Additionally, an experimental setup for bladder volume estimation that does not require imaging was constructed. Finally, experiments were conducted using water-filled balloons as substitutes for the bladder to validate the feasibility of the three-point localization method in measuring bladder volume.

## 2. The “Three-Point Localization” Method

**Figure 1:**
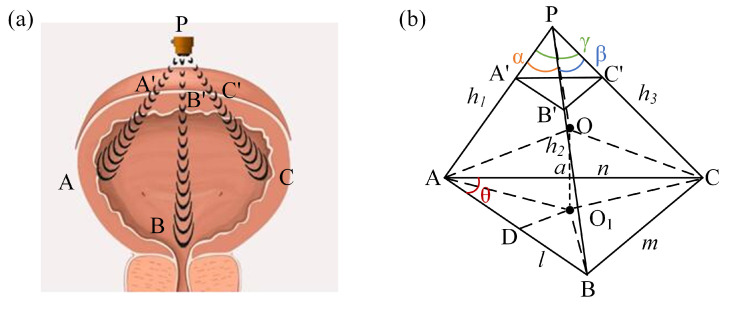
(**a**) Schematic diagram of the “three-point localization” method. (**b**) Triangular prism model.

We propose a “three-point localization” method for bladder volume estimation. Specifically, ultrasound waves can penetrate the bladder wall, and the bladder wall can reflect ultrasound waves. When estimating bladder volume, we approximate the bladder as a sphere [33,34]. The ultrasound probe is positioned directly above the bladder. It emits ultrasound waves in three directions towards the bladder and receives echo signals from the bladder in each direction. By determining the time taken for the echo signal to travel in each direction, we can obtain the distance from the ultrasound probe to the bladder wall in that direction. Ultimately, by combining the distances from the ultrasound probe to the bladder wall in these three directions, the bladder volume can be determined. The schematic diagram of the “three-point localization” method is depicted in Figure 1a. Note that the method is designed solely for fully spherical bladders. The details are as follows.

First, the distance of the ultrasound echo from the ultrasound probe to the bladder in each direction is determined. In the i-th direction, the time interval for the reflection signal from the upper wall of the bladder (first echo signal), from emission to reception, is denoted as ∆*t*′_*i*_. Meanwhile, the time interval for the reflection signal from the lower wall of the bladder (second echo signal) in the same direction, from emission to reception, is denoted as ∆*t*_*i*_. If the propagation speed of ultrasound in the bladder fluid is S, then the distance traveled by the first echo signal in this direction can be expressed as:

(1) $${h^{\prime }}_{i}=S\times \frac{1}{2}{∆t^{\prime }}_{i}$$

The distance traversed by the second echo signal in this direction can be expressed as:

(2) $$h_{i}=S\times \frac{1}{2}{∆t}_{i}$$

where i ≥ 1.

After obtaining the distance of the ultrasound echo from the ultrasound probe to the bladder in each direction, the “three-point localization” method is simplified as shown in Figure 1b. The ultrasound probe is equivalent to one vertex, the three ultrasound beams represent three edges, and the reflection points to three vertices. Consequently, the paths of ultrasound propagation within the bladder can be regarded as a triangular prism model. According to the principles of geometry, a triangular prism can circumscribe a sphere [31,32], so the volume of the bladder can be approximated by the circumscribed sphere volume of this triangular prism.

In this model, the probe is positioned above the bladder, and its distance to the bladder is denoted as h. Assuming that when ultrasound signals are emitted from the probe (designated as point P) towards the bladder in three different directions, the intersections of each direction with the upper wall of the bladder are labeled A′, B′, and C′, and the intersections with the lower wall of the bladder are labeled A, B, and C, respectively. The distance from the ultrasound probe to the lower wall of the bladder represents the transmission distance of the second echo signal. Therefore, the transmission distances of the second echo signal in the three directions are denoted as $h_{1}$, $h_{2}$, and $h_{3}$, corresponding to PA = $h_{1}$, PB = $h_{2}$, and PC = $h_{3}$, respectively. The angles between PA and PB, PB and PC, and PC and PA are denoted as α, β, and γ, respectively.

With AB = l, BC = m, and AC = n, applying the Law of Cosines, we obtain:

(3) $$l^{2}={h_{1}}^{2}+{h_{2}}^{2}-2h_{1}h_{2}\cos\alpha$$

(4) $$m^{2}={h_{2}}^{2}+{h_{3}}^{2}-2h_{2}h_{3}\cos\beta$$

(5) $$n^{2}={h_{3}}^{2}+{h_{1}}^{2}-2h_{3}h_{1}\cos\gamma$$

Within the base triangle, ABC, the intersection of two perpendicular bisectors is the center of the outer circle O_1_, through the point O_1_ for OD⊥AB and intersects the line AB at point D. The bladder can be regarded as the circumscribed sphere of a triangular prism, implying that the distances from the sphere’s center O to points A′, B′, C′, A, B, and C are equal. Furthermore, drawing a line ‘a’ from point O_1_ perpendicular to triangle ABC, we observe that point O lies on line ‘a’, and OA = OB = OC.

A spatial coordinate system is established. Let the coordinates of A and B be (0,0,0) and (l,0,0), respectively, and ∠BAC = θ, then the coordinate of point C is (ncos⁡θ, nsin⁡θ, 0). Let the coordinates of point P be ($x_{p}$, $y_{p}$, $z_{p}$), according to the equation:

(6) $${x_{p}}^{2}+{y_{p}}^{2}+{z_{p}}^{2}={h_{1}}^{2}$$

(7) $${\left(x_{p}-l\right)}^{2}+{y_{p}}^{2}+{z_{p}}^{2}={h_{2}}^{2}$$

(8) $${\left(x_{p}-n\cos\theta \right)}^{2}+{\left(y_{p}-n\sin\theta \right)}^{2}+{z_{p}}^{2}={h_{3}}^{2}$$

The coordinate of point P ($x_{p}$, $y_{p}$, $z_{p}$) can be obtained.

Let the radius of the outer circle of triangle ABC be r, then $r=\frac{m}{2\sin\theta }$. The coordinate of the outer circle center O_1_ is ($\frac{l}{2}$, $\sqrt{r^{2}-\frac{l^{2}}{4}}$, 0).

Let the coordinate of the center of the sphere O be ($\frac{l}{2}$, $\sqrt{r^{2}-\frac{l^{2}}{4}}$, q), where q represents the coordinate of the sphere center O on the z-axis, describing its position along the z-axis in the spatial coordinate system. According to the OP^2^ = (R + h)^2^, we can conclude that:

(9) $$q=\frac{1}{2z_{p}}\left({h_{1}}^{2}-lx_{p}-y_{p}\sqrt{4r^{2}-l^{2}}-2Rh-h^{2}\right)$$

Based on $R^{2}=r^{2}+q^{2}$, we can determine the radius of the bladder sphere to be R. Consequently, the bladder volume (V) is given by:

(10) $$V=\frac{4}{3}\pi R^{3}$$

## 3. Preparation of a Triple-Element Ultrasound Probe

The attenuation of ultrasound waves varies across different organs and tissues, and the energy attenuation of ultrasound is directly proportional to its frequency. To achieve the objectives of ultrasound examinations, varying ultrasound frequencies are applied for different organs and tissues. The standard frequency range for ultrasound examinations typically falls within the range of 2 MHz to 13 MHz [35]. For tissues with greater depth, a lower operating frequency, such as 2~5 MHz, is selected to achieve deeper penetration. Tissues with higher density and greater surface attenuation coefficients require ultrasound frequencies below 2 MHz. When examining small organs or tissues, ultrasound frequencies typically chosen are equal to or greater than 10 MHz. Given the relatively small, fluid-filled nature of the bladder, we selected the ultrasound probe with a central frequency of 3 MHz for this research. 

**Figure 2:**
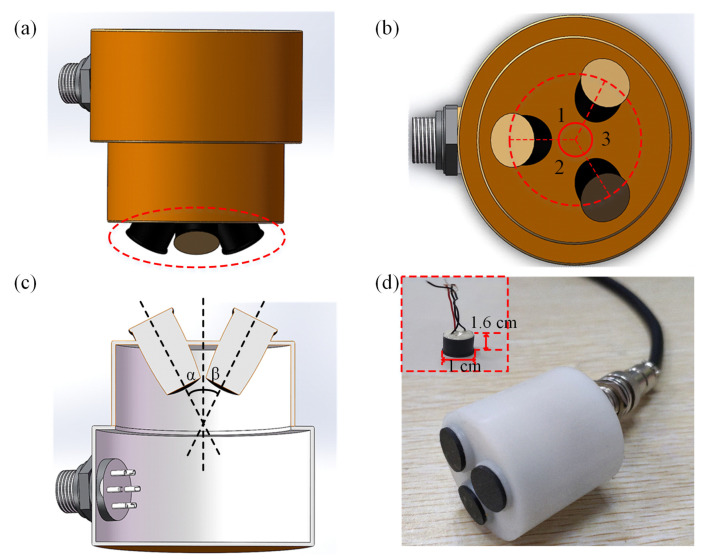
Schematic illustration of the three-element ultrasound probe. (**a**) Front view, (**b**) Top view, and (**c**) Cross-sectional view of the three-element ultrasound probe. (**d**) A physical image of the ultrasound probe and a detailed view of the transducer element.

Based on the three-point localization method, we have designed a corresponding triple-element ultrasound probe. Figure 2a shows a front view of the ultrasound probe, which consists of three ultrasonic transducers, each with the same dimensions. The ultrasonic transducer is composed of the matching layer, the piezoelectric layer (PZT) [23,36,37], and the backing layer E-solder 3022 (Von Roll Isola, New Haven, CT, USA) [38]. Among them, the second matching layer is made of epoxy [38], while the first matching layer consists of epoxy and ZrO_2_. For detailed information on the transducer materials, see Supplementary Table S1. The ultrasound emitting surface is highlighted by the red circle. Furthermore, the top view of the ultrasound probe is presented in Figure 2b, where it can be seen that the centers of the ultrasound array elements are located on the same circumference and ∠1 = ∠2 = ∠3. We can see the cross-section of the ultrasound probe from Figure 2c. Figure 2d displays the physical image of the ultrasound probe. The physical representation of the ultrasound element is illustrated as a red box inside Figure 2d. The structure of a single array element is cylindrical, with specific dimensions of 1 cm in diameter and 1.6 cm in height. The three transducers are driven individually. The characterization results of electrical impedance and pulse-echo testing for the ultrasound transducer are presented in Supplementary Figures S1 and S2, respectively. It can be seen that the central frequency of the transducer is 3 MHz. 

## 4. Experimental Setup

**Figure 3:**
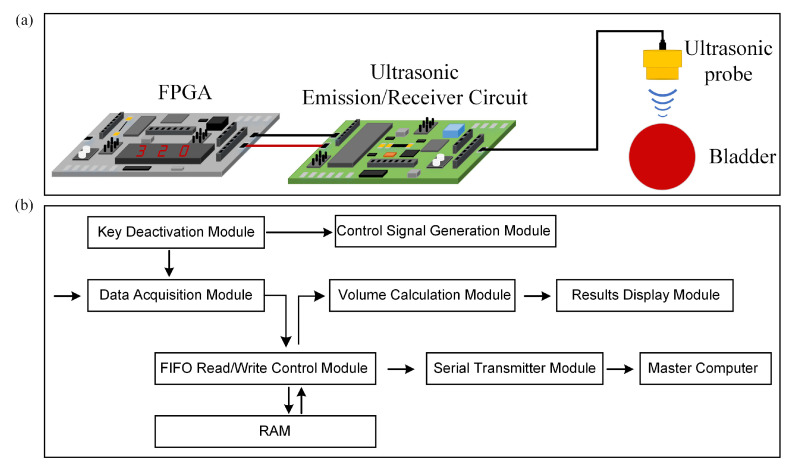
(**a**) Schematic diagram of the experimental setup for bladder volume estimation. (**b**) Overall design of FPGA program.

The experimental setup for bladder volume estimation consists of three main parts: an FPGA development platform, ultrasonic transmission and reception circuits, and a triple-element ultrasound probe. Firstly, the FPGA development platform is employed to generate control signals to drive the ultrasound transmission circuit, store ultrasound echo signals, and compute bladder volume. The core board model of the FPGA development platform is AC7100 (ALINX, Shanghai, China), based on XILINX’s ARTIX-7 series chip XC7A100T-2FFGG484I. The advantages of this core board include a fast processing speed, ample storage capacity, and high bandwidth, making it well-suited for applications such as image processing, high-speed data communication, and rapid data processing. Secondly, the ultrasound transmission and reception circuits play a vital role. The ultrasound transmission circuit generates high-voltage pulse signals of ±90 V to excite the ultrasound probe. The ultrasound reception circuit utilizes a voltage gain of 40 dB to amplify the ultrasound echo signals and then filters out higher-order harmonic components from the echo signals and performs analog-to-digital signal conversion. The ultrasound transmission circuit here mainly consists of two components: MOS field-effect transistors and a driving circuit. The ultrasound reception circuit primarily comprises an analogue-to-digital conversion chip and an amplification and filtering circuit centered around operational amplifiers. Additionally, the direct current (DC) power supply provides power to both the FPGA and the ultrasound transmission and reception circuits. Lastly, there is the ultrasound probe. A schematic diagram of the measurement setup is shown in Figure 3a. In our research, the FPGA development platform was used for signal processing, which allowed us to fully leverage the advantages of FPGA technology, such as its small size, low power consumption, and stable performance, in achieving our research goals of miniaturizing the ultrasound detection system, reducing power consumption, and enhancing accuracy. The design of the FPGA program is depicted in Figure 3b. Control signal generation is determined via an electronic switch. The key debouncing module is responsible for mitigating the impact of electronic switch jitter on the program. Simultaneously, upon generating control signals, the data acquisition module initiates the reception of digital echo signals, writing data into random access memory (RAM) through a FIFO read-write control module. The serial port transmission module sends the data from the RAM to a master computer where the received data is reconstructed into echo signals for validation. Additionally, the volume calculation module computes the bladder volume. The results of the volume calculation are displayed using the result display module.

The FPGA development platform controls the operation of the entire system using the electronic switch integrated into the platform. During the high-level period of the control signal, the ultrasound transmission circuit generates high-voltage pulse signals that can drive the ultrasound probe, causing it to emit ultrasound waves. Ultrasound waves have strong directionality and can propagate in a specific direction [39]. When ultrasound waves encounter surfaces of organ tissues with different acoustic impedances, they undergo reflection, refraction, and scattering [40,41]. Since the ultrasound transmission circuit and ultrasound reception circuit share the same ultrasound probe, ultrasound echoes generate polarities opposite charges on the two opposing surfaces along the polarization direction of the ultrasound probe. This results in a weak voltage signal on the ultrasound probe’s surface through the piezoelectric effect. The ultrasound reception circuit amplifies and filters this small voltage signal and then collects it as a digital signal through an analog-to-digital conversion chip, saving it in the memory of the FPGA. The FPGA performs signal processing on the ultrasound echo in order to extract useful information and finally obtain the volume of the bladder. Note that the key information extracted from the ultrasound echo signals is the peak of these two echo signals. Here, we can extract the signal envelope of the ultrasound signal, which not only simplifies the process of extracting echo signal peaks but also accelerates the operation speed of the FPGA. The envelope extraction algorithm is a mature signal-processing technique that has been applied in various fields. Given the clear and smooth envelope achieved through the Hilbert demodulation method, effectively emphasizing the regularity, periodicity, and frequency traits of the original signal, additional optimization steps are deemed unnecessary. Therefore, in this study, we employ a Hilbert Transform-based demodulation algorithm to extract the signal envelope of ultrasound signals [42].

The definition of the Hilbert Transform is as follows: given a continuous-time signal $x\left(t\right)$ and the impulse response of a linear system denoted as $\hbar =\frac{1}{\pi t}$, the output response, $x_{h}(t),$ of this signal through the linear system is the Hilbert Transform of $x\left(t\right)$. This can be mathematically represented as:

(11) $$H[x\left(t\right)]=x_{h}(t)=\frac{1}{\pi }\int_{-\infty }^{+\infty }\frac{x(\tau )}{x-\tau }d\tau$$

The ultrasound signal is a narrow pulse signal, denoted as s(t), and $\hat{s}(t)$ is its Hilbert Transform. Therefore

(12) $$\left|A\left(t\right)\right|=\sqrt{{{s(t)}^{2}+\hat{s}(t)}^{2}}$$

where $\left|A\left(t\right)\right|$ is the envelope that we require.

## 5. Results and Discussion

**Figure 4:**
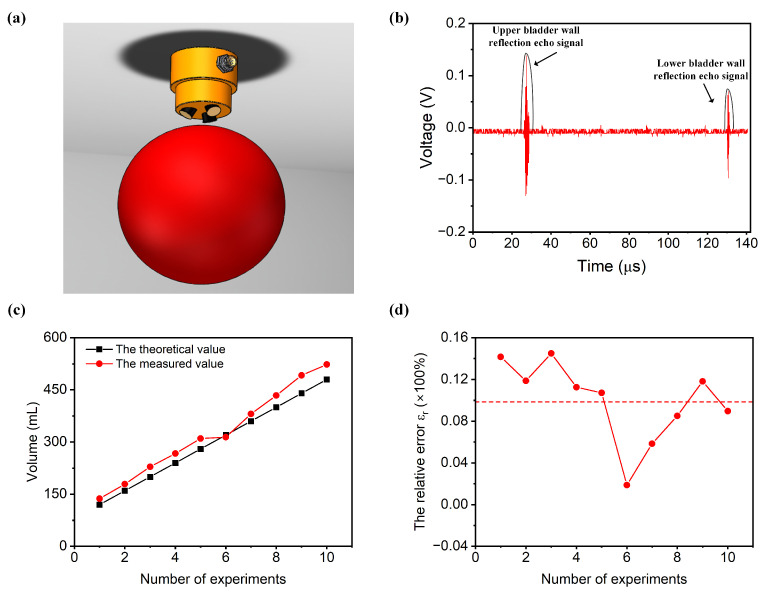
The measurement of bladder volume. (**a**) The experimental schematic diagram for measuring bladder volume using ultrasonic probe. (**b**) The ultrasound echo signal and the envelope of the echo signal. (**c**) The measured values and true values for different bladder volumes. (**d**) The relative error in bladder volume measurement.

The bladder is a vital organ in the human body, located within the abdominal cavity. Its primary function is to store urine. In adults, the bladder typically holds 350 mL to 500 mL of urine, with a maximum capacity of 800 mL. When filled with urine, it assumes a spherical shape, with its radius generally ranging from 7.13 cm to 9.38 cm. Here, we used a water-filled balloon to simulate the real measurement environment of the bladder, as shown in Figure 4a. The angle set between the ultrasound transducers, α = β = γ = 10°, is determined by their geometric considerations. A narrow angle between the transducers might cause the ultrasound beams to run parallel, leading to confusion in their emission and reception. Conversely, if the angle is too wide, it might result in at least one ultrasound transducer emitting waves that do not pass through the bladder, significantly limiting the measurable range of bladder volumes. The ultrasound probe was positioned 2 cm above the balloon and aligned with the center of the balloon, and the balloon was submerged in a water tank. Note that here, the probe transmitted the sound wave by water. We conducted volume measurements on a balloon with a volume of 120 mL. Figure 4b exhibits the ultrasound echo signal and the envelope of the echo signal extracted by the FPGA using the Hilbert Transform demodulation algorithm. The red line represents the echo signal, while the black line illustrates the envelope of the echo signal. Within the envelope signal, we can identify two peak points corresponding to the ultrasound signal. Let us assume that the times of these two peak points are denoted as ${t^{\prime }}_{1}$ and $t_{1}$. Using Equations (1) and (2), we can determine the distance of the ultrasound echo signal from the ultrasound probe to the bladder. With the ultrasound echoes from three different directions, we can obtain three propagation distances: $h_{1}$, $h_{2}$, and $h_{3}$. By employing Equation (3) through Equation (10), we can calculate the bladder volume V. The FPGA will display the specific volume value on a digital tube. In this experiment, the bladder volume was measured to be 135 mL. The entire process, from pressing the electronic switch on the FPGA development platform to displaying the volume, takes a total of 5 s.

Furthermore, we conducted ten tests, sequentially measuring the volumes of balloons with capacities of 120 mL, 160 mL, 200 mL, 240 mL, 280 mL, 320 mL, 360 mL, 400 mL, 440 mL, and 480 mL. Figure 4c presents the results of the measurements compared to the true values for different volumes. The black line in Figure 4c represents the theoretical volume, while the red line represents the experimental measurement. It can be observed that the theoretical and experimental values are in close agreement.

An error analysis of the experimental data was conducted. The absolute error $\varepsilon_{a}$ can be expressed as follows:

(13) $$\varepsilon_{a}=|x-a|$$

where x is the bladder volume measurement value and a is the true bladder volume value.

The relative error $\varepsilon_{r}$ is the percentage ratio of the absolute error to the true value, which can be represented as:

(14) $$\varepsilon_{r}=\varepsilon_{a}/a$$

By substituting the measured values of the bladder volume and the true values into Equations (13) and (14), we can obtain the relative error for each experiment, as shown in Figure 4d. It is clearly observed that the relative error rate stabilizes at 10%, achieving the goal of system accuracy. Currently, the estimation of bladder volume commonly relies on 2D or 3D ultrasound imaging methods. However, the clarity of the bladder contour in ultrasound images can impact the accuracy of bladder volume measurements, and this process is highly dependent on the technical proficiency of the operator. Additionally, the cost and size of ultrasound probes used for 3D imaging are substantial. In contrast, the ultrasonic measurement method proposed in this study does not require imaging, significantly reduces the instrument size, lowers the costs, and shortens the testing time. 

The experiment we conducted did not consider the impact of the human abdominal adipose tissue on the measurements. The bladder, located beneath the abdomen, is insulated from the skin surface by a layer of fat, which significantly attenuates ultrasound waves. In our subsequent research, we plan to introduce a layer of material between the ultrasound probe and the balloon that can simulate the impact of the fat layer on ultrasound, thereby further enhancing the experimental design. Additionally, bladder volume is subject to individual variations, and different individuals may exhibit different conditions. When a patient’s bladder volume is excessively large, the bladder volume estimation method proposed in this paper may introduce significant measurement errors. We will conduct further studies in our subsequent work to meet the needs of a more diverse patient population. In this study, we mainly provide a feasible method to measure bladder volume

## 6. Conclusions

In summary, we introduced an innovative three-point localization method for calculating bladder volume. Based on the proposed method, we designed a matching three-element ultrasound probe with a central frequency of 3 MHz, achieving the objective of a single probe emitting ultrasound waves in multiple directions. Utilizing an FPGA development platform for signal processing allowed us to fully leverage the advantages of FPGA technology, including its small form factor, low power consumption, and stable performance. Additionally, the Hilbert Transform-based demodulation algorithm was used to extract the envelope of the ultrasound signal, which reduces the difficulty and accelerates the speed of signal processing. The measurement results for different bladder volumes indicate that the three-point localization method can accurately measure bladder volume, with a stable relative error rate of 10% and a measurement time of under 5 s. In conclusion, this work preliminarily investigated the viability of the three-point localization method for estimating bladder volume. 

## Data Availability

Data are contained within the article and Supplementary Materials.

## Supplementary Materials

The following supporting information can be downloaded at: https://www.mdpi.com/article/10.3390/s24061932/s1, Figure S1: The electrical impedance test of transducer; Figure S2: (a) The echo signal of the transducer. (b) The frequency gain diagram of the echo signal; Table S1: Transducer Material Parameters.

## References

[1] Minassian V.A., Drutz H.P., Al-Badr A. (2003). Urinary incontinence as a worldwide problem. Int. J. Gynecol. Obstet..

[2] Öztürk R., Murt A. (2020). Epidemiology of urological infections: A global burden. World J. Urol..

[3] Li L., Wu J., Lyon C.J., Jiang L., Hu T.Y. (2023). Clinical peptidomics: Advances in instrumentation, analyses, and applications. BME Front..

[4] Tannenbaum C., Perrin L., DuBeau C.E., Kuchel G.A. (2001). Diagnosis and management of urinary incontinence in the older patient. Arch. Phys. Med. Rehabil..

[5] Kim A., Powell C.R., Ziaie B. (2014). An implantable pressure sensing system with electromechanical interrogation scheme. IEEE Trans. Biomed. Eng..

[6] Kim M.K., Kim H., Jung Y.S., Adem K.M.A., Bawazir S.S., Stefanini C., Lee H.J. Implantable bladder volume sensor based on resistor ladder network composed of conductive hydrogel composite. Proceedings of the 2017 39th Annual International Conference of the IEEE Engineering in Medicine and Biology Society (EMBC).

[7] Stauffer F., Zhang Q., Tybrandt K., Zambrano B.L., Hengsteler J., Stoll A., Trüeb C., Hagander M., Sujata J.-M., Hoffmann F. (2018). Soft electronic strain sensor with chipless wireless readout: Toward real-time monitoring of bladder volume. Adv. Mater. Technol..

[8] Chen S.C., Hsieh T.H., Fan W.J., Lai C.H., Chen C.L., Wei W.F., Peng C.W. (2015). Design and evaluation of potentiometric principles for bladder volume monitoring: A preliminary study. Sensors.

[9] Waltz F.M., Timm G.W., Bradley W.E. (1971). Bladder volume sensing by resistance measurement. IEEE Trans. Biomed. Eng..

[10] Rajagopalan S., Sawan M., Ghafar-Zadeh E., Savadogo O., Chodavarapu V.P. (2008). A polypyrrole-based strain sensor dedicated to measure bladder volume in patients with urinary dysfunction. Sensors.

[11] Melgaard J., Rijkhoff N.J.M. (2011). Detecting the onset of urinary bladder contractions using an implantable pressure sensor. IEEE Trans. Neural Syst. Rehabil. Eng..

[12] Lee D.S., Kim S.J., Sohn D.W., Choi B., Lee M.K., Lee S.J., Kim S.W. (2011). Real-time bladder volume monitoring by the application of a new implantable bladder volume sensor for a small animal model. Kaohsiung J. Med. Sci..

[13] Wang J., Hou C., Zheng X., Zhang W., Chen A., Xu Z. (2009). Design and evaluation of a new bladder volume monitor. Arch. Phys. Med. Rehabil..

[14] Schurger A., Sitt J.D., Dehaene S. (2012). An accumulator model for spontaneous neural activity prior to self-initiated movement. Proc. Natl. Acad. Sci. USA.

[15] Majerus S.J., Garverick S.L., Suster M.A., Fletter P.C., Damaser M.S. (2012). Wireless, ultra-low-power implantable sensor for chronic bladder pressure monitoring. ACM J. Emerg. Technol. Comput. Syst..

[16] Leonhardt S., Cordes A., Plewa H., Pikkemaat R., Soljanik I., Moehring K., Gerner H.J., Rupp R. (2011). Electric impedance tomography for monitoring volume and size of the urinary bladder. Biomed. Tech..

[17] Li R., Gao J., Li Y., Wu J., Zhao Z., Liu Y. (2016). Preliminary study of assessing bladder urinary volume using electrical impedance tomography. J. Med. Biol. Eng..

[18] Noyori S.S., Nakagami G., Sanada H. (2022). Non-invasive urine volume estimation in the bladder by electrical impedance-based methods: A review. Med. Eng. Phys..

[19] Gong Q.Y., Tan L.T., Romaniuk C.S., Jones B., Brunt J.N., Roberts N. (1999). Determination of tumour regression rates during radiotherapy for cervical carcinoma by serial MRI: Comparison of two measurement techniques and examination of intraobserver and interobserver variability. Br. J. Radiol..

[20] Heverhagen J.T., Hartlieb T., Boehm D., Klose K.J., Wagner H.J. (2002). Magnetic resonance cystometry: Accurate assessment of bladder volume with magnetic resonance imaging. Urology.

[21] Ma Z., Jorge R.N., Mascarenhas T., Tavares J.M.R. (2011). Novel approach to segment the inner and outer boundaries of the bladder wall in T_2_-weighted magnetic resonance images. Ann. Biomed. Eng..

[22] Gawthrop J., Oates R. (2012). Measured bladder volume for radiotherapy of the prostate using the hand-held BladderScan^®^ BVI 3000. Radiographer.

[23] Li J., Ma Y., Zhang T., Shung K.K., Zhu B. (2022). Recent Advancements in Ultrasound Transducer: From Material Strategies to Biomedical Applications. BME Front..

[24] Zhang L., Marcus C., Lin D., Mejorado D., Schoen S.J., Pierce T.T., Kumar V., Fernandez S.V., Hunt D., Li Q. (2023). A conformable phased-array ultrasound patch for bladder volume monitoring. Nat. Electron..

[25] Deng X., Xu T., Huang G., Li Q., Luo L., Zhao Y., Wu Z., Ou-Yang J., Yang X., Xie M. (2020). Design and fabrication of a novel dual-frequency confocal ultrasound transducer for microvessels super-harmonic imaging. IEEE Trans. Ultrason. Ferroelectr. Freq. Control.

[26] Li J., Wang Z., Jiang L., Yu Z., Ge X., Ouyang J., Yang X., Tian X., Tian H., Zhu B. (2023). High Efficiency and Anomalous Photoacoustic Behavior in Vertical CNTs Array. Energy Environ. Mater..

[27] Jiang L., Lu G., Yang Y., Xu Y., Qi F., Li J., Zhu B., Chen Y. (2021). Multichannel piezo-ultrasound implant with hybrid waterborne acoustic metastructure for selective wireless energy transfer at megahertz frequencies. Adv. Mater..

[28] McLean G.K., Edell S.L. (1978). Determination of bladder volumes by gray scale ultrasonography. Radiology.

[29] Baek J., Huh J., Kim M., Hyun An S., Oh Y., Kim D., Lee R. (2013). Accuracy of volume measurement using 3D ultrasound and development of CT-3D US image fusion algorithm for prostate cancer radiotherapy. Med. Phys..

[30] Niu H., Yang S., Liu C., Yan Y., Li L., Ma F., Wang X., Pu F., Li D., Fan Y. Design of an ultrasound bladder volume measurement and alarm system. Proceedings of the International Conference On Bioinformatics and Biomedical Engineering.

[31] Donnay J.D. (2011). Spherical Trigonometry.

[32] Apostol T.M., Mnatsakanian M.A. (2006). Solids circumscribing spheres. Am. Math. Mon..

[33] Sánchez J.D.M., Leyton V.H.M., Rodas C.F.R. Effect of the injection and measurement patterns and the geometric distribution of electrodes in bladder volume estimation using electrical impedance tomography. Proceedings of the 2021 XXIII Symposium on Image, Signal Processing and Artificial Vision (STSIVA).

[34] Li K., Guo Q., Guo J. (2017). Novel algorithms for reducing bladder volume estimation error caused by scanning positions. Int. J. Comput. Math..

[35] Sedlaczek J., Lohmann C.H., Lotz E.M., Hyzy S.L., Boyan B.D., Schwartz Z. (2017). Effects of low-frequency ultrasound treatment of titanium surface roughness on osteoblast phenotype and maturation. Clin. Oral Implant. Res..

[36] Jain A., Prashanth K.J., Sharma A.K., Jain A., Rashmi P.N. (2015). Dielectric and piezoelectric properties of PVDF/PZT composites: A review. Polym. Eng. Sci..

[37] Kahn M. (1985). Acoustic and elastic properties of PZT ceramics with anisotropic pores. J. Am. Ceram. Soc..

[38] Zhou Q., Lam K.H., Zheng H., Qiu W., Shung K.K. (2014). Piezoelectric single crystal ultrasonic transducers for biomedical applications. Prog. Mater. Sci..

[39] Bi X. (2021). Infrared Sensors and Ultrasonic Sensors. Environmental Perception Technology for Unmanned Systems.

[40] Jambrak A.R., Lelas V., Mason T.J., Krešić G., Badanjak M. (2009). Physical properties of ultrasound treated soy proteins. J. Food Eng..

[41] Jung U., Choi H. (2022). Active echo signals and image optimization techniques via software filter correction of ultrasound system. Appl. Acoust..

[42] Assef A.A., Ferreira B.M., Maia J.M., Costa E.T. (2018). Modeling and FPGA-based implementation of an efficient and simple envelope detector using a Hilbert Transform FIR filter for ultrasound imaging applications. Res. Biomed. Eng..

