# Supplementary material for: A Novel Three-Point Localization Method for Bladder Volume Estimation
Source: Sensors (Basel). 2024 Mar 18;24(6):1932. doi: 10.3390/s24061932 (PMC10975550; doi:10.3390/s24061932)
Supplement: Supplementary file 1 [file sensors-24-01932-s001.zip › sensors-2765245-supplementary.pdf]

A novel three-point localization method for bladder volume estimation

Junru Yuan, Mingke Shen, Tao Zhang, Jun Ou-Yang, Xiaofei Yang, Benpeng Zhu*

School of Integrated Circuits, Huazhong University of Science and Technology, Wuhan 430074, China.

*Author to whom correspondence should be addressed.

E-mail: benpengzhu@hust.edu.cn

Supplementary Materials

Transducer Material Parameters Table S1

Material	Function	Density (kg/m^3)	Velocity(m/s)	Thicknesses(μm)
Epoxy	The second matching layer	1150	2650	190
Epoxy+ZrO_2	The first matching layer	2430	3200	240
PZT	The piezoelectric layer	7500	4400	540
E-solder 3022	The backing layer	3200	1850	5000

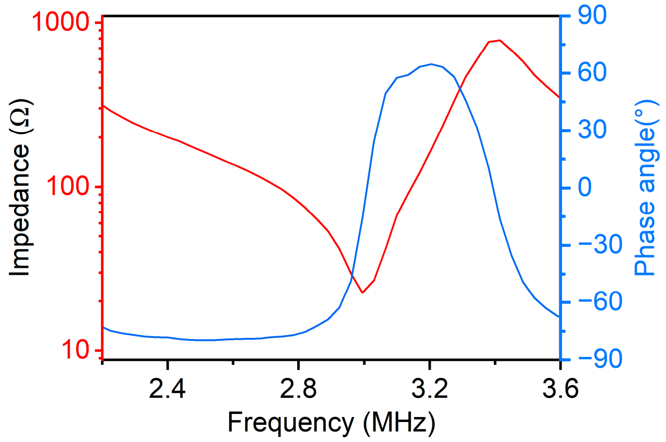

Figure S1. The electrical impedance test of transducer.

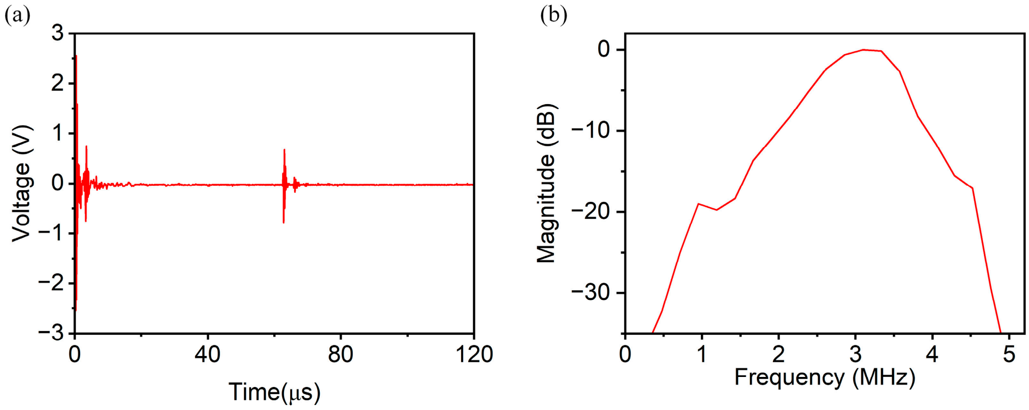

Figure S2. (a) The echo signal of the transducer. (b) The frequency gain diagram of the echo signal.
